# A mini review of plan quality and secondary cancer risk in CyberKnife M6 radiosurgery for benign intracranial tumors

**DOI:** 10.3389/fonc.2024.1453256

**Published:** 2024-08-07

**Authors:** Pei-Ju Chao, Tsair-Fwu Lee

**Affiliations:** ^1^ Medical Physics and Informatics Laboratory of Electronics Engineering, National Kaohsiung University of Science and Technology, Kaohsiung, Taiwan; ^2^ Department of Radiation Oncology, Kaohsiung Chang Gung Memorial Hospital and Chang Gung University College of Medicine, Kaohsiung, Taiwan; ^3^ Graduate Institute of Clinical Medicine, Kaohsiung Medical University, Kaohsiung, Taiwan; ^4^ Department of Medical Imaging and Radiological Sciences, Kaohsiung Medical University, Kaohsiung, Taiwan; ^5^ School of Dentistry, College of Dental Medicine, Kaohsiung Medical University, Kaohsiung, Taiwan

**Keywords:** stereotactic radiosurgery (SRS), CyberKnife M6, multileaf collimator (MLC), iris collimator, secondary cancer risk (SCR), treatment planning

## Abstract

With advancements in medical technology, stereotactic radiosurgery (SRS) has become an essential option for treating benign intracranial tumors. Due to its minimal side effects and high local control rate, SRS is widely applied. This paper evaluates the plan quality and secondary cancer risk (SCR) in patients with benign intracranial tumors treated with the CyberKnife M6 system. The CyberKnife M6 robotic radiosurgery system features both multileaf collimator (MLC) and IRIS variable aperture collimator systems, providing different treatment options. The study included 15 patients treated with the CyberKnife M6 system, examining the differences in plan quality and SCR between MLC and IRIS systems. Results showed that MLC and IRIS plans had equal PTV (planning target volume) coverage (98.57% vs. 98.75%). However, MLC plans demonstrated better dose falloff and conformity index (CI: 1.81 ± 0.26 vs. 1.92 ± 0.27, P = 0.025). SCR assessment indicated that MLC plans had lower cancer risk estimates, with IRIS plans having average LAR (lifetime attributable risk) and EAR (excess absolute risk) values approximately 25% higher for cancer induction and 15% higher for sarcoma induction compared to MLC plans. The study showed that increasing tumor volume increases SCR probability, but there was no significant difference between different plans in PTV and brainstem analyses.

## Introduction

With advancements in medical technology, stereotactic radiosurgery (SRS) has become an essential option for treating benign intracranial tumors. SRS is widely used due to its minimal side effects and good local control rates. The CyberKnife M6 robotic radiosurgery system is an advanced SRS device that offers different treatment options through its multileaf collimator (MLC) and IRIS variable aperture collimator systems (1–3). This study evaluates the plan quality and secondary cancer risk (SCR) in patients with benign intracranial tumors treated with the CyberKnife M6 system (4).

Despite the significant success of SRS in treating benign intracranial tumors, SCR remains a critical concern, particularly for younger patients. Radiotherapy can expose healthy tissues to unnecessary radiation doses, increasing the risk of SCR. Therefore, it is necessary to compare different radiotherapy techniques to ensure optimal treatment outcomes while minimizing long-term risks. This study aims to address several core questions:

1. What are the differences in plan quality between MLC and IRIS collimator systems in the CyberKnife M6 system?

2. How do these systems impact the radiation dose to healthy tissues when treating benign intracranial tumors?

3. What are the differences in SCR assessment between MLC and IRIS systems?

4. Which technology can provide effective treatment while minimizing SCR?

The significance of this study lies in several aspects:

1. Clinical Application Guidance: The results of this study will provide essential references for clinicians in selecting radiotherapy plans, helping them choose the optimal treatment technique based on specific patient conditions.
2. Patient Safety: Understanding the SCR differences between different technologies helps develop safer treatment plans, reducing long-term risks for patients, especially younger ones.
3. Technological Improvement: Comparing the advantages and disadvantages of different collimator systems can promote further advancements and innovations in radiotherapy technology, enhancing overall treatment levels.
4. Scientific Research Foundation: The data and analysis provided by this study will lay the foundation for future related research, driving further development in the field of radiotherapy.

## Literature review

SRS is a highly precise radiotherapy technique that can concentrate high doses of radiation on the tumor area while minimizing damage to surrounding healthy tissues. Previous studies have shown that SRS has significant local control rates and low side effects in treating benign intracranial tumors and brain metastases (5–7). Additionally, SRS is used as an adjunct to surgical resection to enhance treatment outcomes. However, long-term SCR remains a key consideration for younger patients. Studies indicate that peripheral dose (PD) is an essential factor affecting SCR, primarily composed of scatter radiation from the treatment machine head and collimators (8–10). Factors influencing PD include collimator type, monitor units (MU), collimator size, number of beams, and beam angles. According to Delaby et al., PD during brain SRS with the CyberKnife M6 system is approximately 0.06% of MU with a 20 mm fixed collimator and 0.04% with an IRIS collimator (11).

Plan quality evaluation typically includes dosimetric indices such as planning target volume (PTV) coverage, conformity index (CI), and dose gradients (R10% and R50%). Studies have found that MLC plans have better dose gradients and lower SCR compared to IRIS plans. Jang et al. reported that MLC plans had slightly higher CI values in single and multiple brain metastases patients compared to IRIS plans (12). SCR assessment usually employs the organ equivalent dose (OED) concept and excess absolute risk (EAR) analysis. Schneider et al.’s model considers the effects of repopulation, proliferation, and cell killing, while the BEIR VII model, based on epidemiological data from Hiroshima atomic bomb survivors, is also used for comparison (2, 13). Overall, SRS’s application in intracranial tumor treatment has been extensively studied and proven effective. However, SCR for younger patients requires careful evaluation. Choosing the appropriate collimator system and optimizing radiotherapy plans can improve treatment outcomes while reducing long-term risks. Future research should focus on larger patient cohorts and long-term follow-up to further validate these findings.

## Methods

### Patient data

The study included 15 patients with benign intracranial tumors treated with the CyberKnife M6 system (4). Each patient’s tumor had a median volume of 6.43 cm³ (range 0.33-29.72 cm³), and the age range was 15 to 67 years (median 54 years). All patients received treatment using either the MLC or IRIS collimator system. The diverse age range and tumor volume help assess the CyberKnife M6 system’s efficacy and risks across different ages and tumor sizes. However, the sample size of only 15 patients may not provide statistically significant conclusions, suggesting the need for larger samples in future studies to enhance reliability and generalizability.

### Treatment planning

Treatment plans were created using the Multiplan treatment planning software (version 5.1.3). LightSpeed RT16 CT simulation scans and MRI image fusion were used to delineate the planning target volume (PTV), ensuring precise dose delivery to the tumor area. Applying a minimum dose to cover >95% of the target is a reasonable treatment goal, ensuring treatment efficacy while maximizing the protection of surrounding healthy tissues. These methods comply with clinical standards, providing accurate dosimetric data.

### Dosimetric evaluation

Evaluating plan quality using dosimetric indices like PTV coverage and the conformity index (CI) is a common and effective method. These calculations enable a quantitative assessment of a treatment plan’s efficacy and safety. The indices help to discern performance differences between collimator systems, providing a basis for optimizing treatment plans. However, these evaluations mainly focus on the geometric characteristics of dose distribution and do not consider biological responses. Future studies should incorporate biological dosimetry assessments for a more comprehensive evaluation of plan quality.

In this study, the conformity index (CI) is calculated using the definition: CI = PIV/TIV, where TIV is the target isodose volume, and PIV is the volume of treatment with the prescribed isodose. This definition assesses how well the prescription dose conforms to the target volume, indicating the precision of dose delivery to the target area. Additionally, coverage is defined as Coverage = TIV/PTV, with TIV being the target isodose volume and PTV being the planning target volume.

For gradient indices, we used the following definitions: R50% is defined as the ratio of the volume receiving 50% of the prescribed dose (V50%) to the PTV, and R10% is defined as the ratio of the volume receiving 10% of the prescribed dose (V10%) to the PTV. These indices measure the dose distribution gradient and falloff in the treatment plans, ensuring the dosimetric quality and effectiveness of the radiation therapy.

### Secondary cancer risk assessment

Using Schneider’s and BEIR VII models for SCR assessment is a reasonable choice (13, 14). These models consider different dose-response relationships and provide cancer risk estimates. Extracting dose-volume histogram (DVH) data and applying these models can quantify SCR for different treatment plans. However, these models are based on assumptions and statistical data, which may contain uncertainties. It is recommended to consider model limitations when evaluating results and, if possible, conduct long-term follow-up to verify these risk estimates. Overall, the methodology is suitable for assessing plan quality and SCR in treating benign intracranial tumors with the CyberKnife M6 system. However, the small sample size suggests the need for larger-scale studies in the future to enhance the reliability and generalizability of results. Combining biological dosimetry assessments and long-term follow-up will help provide a more comprehensive risk assessment.

## Results

### Dosimetric results

The dosimetric analysis presented in **Table 1** reveals the comparative effectiveness of MLC and IRIS plans in CyberKnife radiosurgery, highlighting their implications for secondary cancer risk (SCR) management. Both MLC and IRIS plans show similar PTV coverage, with 98.57% for MLC and 98.75% for IRIS, indicating effective tumor coverage by both techniques. However, MLC plans demonstrate superior performance in dose falloff, evidenced by lower R50% and R10% values. This suggests more rapid dose attenuation around the tumor, providing better protection for surrounding healthy tissues.

**Table 1:** Comparative analysis of dosimetric quality and SCR between MLC and IRIS plans in cyberKnife radiosurgery.

Plan Type	PTV Coverage (%)	Coverage Ratio (MLC/IRIS)	Conformity Index (CI)	P-Value (CI)	R50% (Mean ± SD, Median)	R10% (Mean ± SD, Median)	R50% Ratio (MLC/IRIS)	R10% Ratio (MLC/IRIS)
MLC	98.57	0.9981	1.81 ± 0.26	0.025	4.64 ± 1.20, 4.56	54.99 ± 28.92, 45.56	0.84	1.02
IRIS	98.75		1.92 ± 0.27		5.52 ± 1.41, 5.74	54.00 ± 25.60, 45.74		

data represent mean ± SD (median); With a p-value of 0.05 from the Wilcoxon Signed Ranked Test; R50%, which is represented by the ratio of the volume covered by the 50% prescription isodose line of the maximum target dose (D50%) to the target volume; R10%, which is represented by the ratio of the volume covered by the 10% prescription isodose line of the maximum target dose (D10%) to the target volume.

MLC, multi-leaf collimator; IRIS, Iris collimator; EAR, Excess Absolute Risk; CI, conformal index.

Additionally, the Conformity Index (CI) analysis further distinguishes the two, showing that MLC plans conform more accurately to the tumor shape with a CI of 1.81 ± 0.26, compared to IRIS plans at 1.92 ± 0.27, with the difference being statistically significant (P = 0.025). This indicates that MLC plans not only target the tumor more precisely but also minimize radiation exposure to adjacent healthy tissues more effectively.

**Table 1** emphasizes these dosimetric advantages, with the lower CI values in MLC plans reflecting their enhanced precision and reduced exposure to surrounding tissues, a critical factor in minimizing SCR. The lower R50% values for MLC plans underscore their superior high-dose containment within the target, an advantage that is quantitatively supported by the R50% and R10% ratios and confirmed by significant Wilcoxon Signed-Rank p-values.

Overall, this integrated analysis underscores the superiority of MLC plans in terms of both high-dose control and conformity, making them a safer option for radiosurgery. The insights derived from **Table 1** are crucial for clinicians aiming to optimize treatment strategies and enhance patient safety, offering better control over radiation doses with a comparable spread of low doses.

### OAR dose commentary

This study analyzed the dose impact on OAR during radiosurgery for benign intracranial tumors using the CyberKnife M6 system. It found significant differences in the dose impacts on various OARs between MLC and IRIS plans. For instance, the optic chiasm received lower doses in MLC plans with average and maximum doses of 0.91 Gy and 2.09 Gy respectively, compared to 1.41 Gy and 2.76 Gy in IRIS plans. Similarly, the right optic nerve and brainstem also showed reduced dose impacts in MLC plans, emphasizing its better shielding effect compared to IRIS. In terms of soft tissue, while the average doses were lower in MLC plans (1.31 Gy vs. 1.46 Gy in IRIS), the maximum doses were slightly higher (18.51 Gy vs. 18.31 Gy), showing a negligible difference.

### Dose gradient effects and clinical implications

The dose gradient effects, assessed through R50% and R10%, underscored the superior performance of MLC plans in controlling high-dose regions. The R50% values were 4.64 ± 1.20 for MLC and 5.52 ± 1.41 for IRIS, with the MLC/IRIS ratio at 0.84 indicating a better dose fall-off in high-dose areas (P < 0.05). Conversely, R10% values showed no significant difference between the plans, with MLC achieving 54.99 ± 28.92 compared to IRIS’s 54.00 ± 25.60, reinforcing that both plans managed low-dose spread equally well.

### Secondary cancer risk

SCR assessment showed that MLC plans had lower cancer risk estimates across various SCR models. Specifically, IRIS plans had average LAR (lifetime attributable risk) and EAR (excess absolute risk) values approximately 25% higher for cancer induction and 15% higher for sarcoma induction compared to MLC plans. This indicates that, despite similar PTV coverage and CI between the two plans, MLC plans more effectively reduce long-term SCR. The study also showed that increasing tumor volume increases SCR probability, but there was no significant difference between different plans in PTV and brainstem analyses, suggesting that SCR is mainly influenced by tumor volume rather than the plan type.

These findings indicate that, despite similar PTV coverage between MLC and IRIS plans, MLC plans perform better in dose falloff and conformity, effectively reducing long-term SCR. This is significant for clinical practice, as choosing the appropriate collimator system can ensure treatment effectiveness while significantly reducing long-term health risks, especially for younger patients.

### Impact of lesion size/treatment size on SCR in stereotactic radiosurgery

Using the CyberKnife M6 robotic radiosurgery system, this study explored how lesion size and treatment volume impact SCR, analyzing the dosimetric quality of different plans (MLC and IRIS) and their implications.

a. Impact of Lesion Size on SCR: The research findings indicate that larger tumor volumes are directly correlated with an increased probability of SCR. Although no significant differences were noted between the MLC and IRIS plans in treating the planning target volume (PTV) and brainstem, significant differences in excess absolute risk (EAR) were observed in soft tissues, particularly with tumor volumes ranging from 1–10 cc and exceeding 10 cc.
b. Impact of Treatment Plans on SCR: Using the Schneider and BEIR VII models to assess SCR risks, the study found that the IRIS plan exhibited average lifetime attributable risk (LAR)/EAR values approximately 25% higher for cancer induction and 15% higher for sarcoma induction than the MLC plan. The MLC plan demonstrated superior dose gradient and conformity, suggesting a lower risk of SCR. Notably, the EAR/LAR values for the IRIS plan were slightly higher than those for the MLC plan across all patients, especially in soft tissues.
c. Key findings from Supplementary Figures S1, S2 in (4): These figures underscore the substantial effects that lesion size and treatment modalities (MLC vs. IRIS) have on SCR in patients undergoing treatment with the CyberKnife M6 system. They detail how the organ equivalent dose (OED)/EAR and OED/lifetime attributable risk (LAR) values vary with patient age, treatment size, and targeted organs, emphasizing that younger patients and larger treatment sizes generally lead to increased SCR. These insights are invaluable for refining treatment plans, aiming to enhance patient safety and efficacy in radiosurgical strategies.

This synthesis highlights the intricate relationship between treatment strategy, lesion size, and the consequent risk of secondary cancers, illustrating the necessity of careful planning to mitigate long-term risks while maximizing the therapeutic outcomes of radiosurgery.

## Discussion

The study demonstrates that MLC plans of the CyberKnife M6 system are superior to IRIS plans in terms of dosimetric quality and SCR reduction. MLC plans offer better conformity and dose gradients, making them more effective at reducing SCR, particularly in cases with increasing target volume—a factor that significantly elevates SCR risks and should be carefully considered in plan design.

Moreover, MLC plans not only reduce doses to OAR more effectively than IRIS plans, especially in high-dose gradients, but also potentially lower SCR risks. The advantages of MLC plans, including refined control over radiation doses and enhanced protection of critical structures, make them the preferred choice in clinical settings. These results underscore the significant impact that both lesion size and treatment plans have on SCR. The superior dose gradient and conformity of the MLC plans contribute to their effectiveness in mitigating these risks, offering vital insights for the clinical selection and optimization of radiotherapy plans.

More research should focus on validating these findings through studies with larger sample sizes and extended follow-ups. This will not only optimize radiotherapy strategies and improve treatment outcomes for patients with intracranial tumors but will also help to substantiate these results and provide more comprehensive clinical guidance.

### Current state of related research

In discussing the current state of related research, it is necessary to consider various radiotherapy technologies, dosimetric indices, SCR assessment methods, and their impact on the treatment of benign intracranial tumors.

1. Development of Radiotherapy Technologies: Radiotherapy technology is continually evolving, and SRS has become an important method for treating benign intracranial tumors (15). The CyberKnife M6 robotic radiosurgery system, known for its high precision and flexibility, is an advanced device in SRS. Other technologies like MLC and IRIS collimator systems are also widely used clinically. Studies have shown that these technologies significantly improve treatment outcomes and reduce doses to healthy tissues.
2. Dosimetric Indices and Plan Quality Assessment: Dosimetric indices are crucial standards for assessing radiotherapy plan quality. Indices like PTV coverage, conformity index (CI), and dose gradients (R10% and R50%) are used to evaluate the accuracy and effectiveness of treatment plans (16, 17). Literature indicates that MLC plans perform better than IRIS plans in terms of conformity and dose gradients. Additionally, MLC plans’ superior dose falloff helps protect surrounding healthy tissues and reduce side effects.
3. Secondary Cancer Risk (SCR) Assessment: SCR is a significant consideration in radiotherapy. Schneider and BEIR VII models are commonly used for SCR assessment. Studies have shown that MLC plans exhibit lower cancer risk estimates across SCR models. However, SCR assessment still has uncertainties, requiring further research to improve dose-response models (18).
4. Comparison of Different Radiotherapy Technologies: Besides MLC and IRIS technologies, other radiotherapy technologies like proton therapy and volumetric modulated arc therapy (VMAT) are also applied clinically. Proton therapy, with its superior dose distribution characteristics, better protects healthy tissues and has shown potential in reducing SCR in some studies. However, the high cost and equipment requirements limit its widespread application (19).
5. Long-term Follow-up Studies: Most current SCR studies rely on short-term data, lacking long-term follow-up. Since SCR usually manifests years after treatment, future studies need long-term follow-up to comprehensively evaluate the long-term risks and effects of different treatment technologies.

### Future research directions

Based on the study results, future research can delve deeper into the following aspects:

1. Expanding Sample Size: The study included a small sample size (15 patients). Future research should expand the sample size to improve the statistical significance and reliability of results. Additionally, including more age groups and different types of benign intracranial tumors will ensure the generalizability of the results.
2. Long-term Follow-up: Since SCR usually requires a long time to manifest, future studies should conduct long-term follow-up to monitor the long-term health status of patients treated with the CyberKnife M6 system. This will help accurately assess the long-term effects and potential risks of radiotherapy.
3. Comparing Different Radiotherapy Technologies: Besides MLC and IRIS technologies, future research should compare other advanced radiotherapy technologies like proton therapy and VMAT to assess their performance in plan quality and SCR. This will help identify the best treatment options to maximize treatment effectiveness and minimize risks.
4. Improving Dose-response Models: Current dose-response models have some uncertainties. Future research should aim to improve these models, especially for high-dose and non-uniform dose distributions. Combining biologically effective dose (BED) and more precise cellular-level response data will enhance the accuracy of SCR estimates.
5. Multimodal Treatment Combinations: Considering the pros and cons of different treatment modes, future research should explore combining radiotherapy with other treatments like surgery and drug therapy to achieve better treatment outcomes. Studies should include evaluating the impact of these multimodal treatments on SCR and other adverse reactions.
6. Application of Machine Learning and Artificial Intelligence: With the rapid development of data science and artificial intelligence, future research can use machine learning algorithms to optimize radiotherapy plans. By analyzing large amounts of patient data, machine learning can help identify optimal treatment parameters, further improving treatment effectiveness and reducing risks.

These future research directions will not only improve the effectiveness of radiotherapy for benign intracranial tumors but also reduce treatment-related long-term risks, ultimately improving overall patient prognosis and quality of life.

## Conclusion

The study results show that MLC plans have better performance in plan quality and reducing secondary cancer risk (SCR). Although IRIS plans have some advantages in treating small targets, overall, MLC plans are the superior choice. Specifically, MLC plans outperform IRIS plans in conformity index (CI), dose gradients (R50% and R10%), and SCR assessment.

This study provides valuable references for selecting radiotherapy plans, particularly recommending the use of MLC plans when treating benign intracranial tumors. This not only ensures treatment effectiveness but also significantly reduces long-term health risks, especially for younger patients.

Overall, the study emphasizes the advantages of using the MLC collimator system and recommends prioritizing MLC plans when treating benign intracranial tumors. Future research should continue exploring the performance of different plans in larger sample sizes and long-term follow-ups to further validate these findings and provide more comprehensive clinical guidance.

## Author contributions

P-JC: Conceptualization, Methodology, Supervision, Validation, Writing – review & editing, Project administration. T-FL: Conceptualization, Supervision, Validation, Writing – review & editing, Methodology, Writing – original draft, Funding acquisition.

## References

[1] Kathriarachchi V Shang C Evans G Leventouri T Kalantzis G. Dosimetric and radio biological comparison of CyberKnife M6™ InCise multileaf collimator over IRIS™ variable collimator in prostate stereotactic body radiation therapy. J Med Phys. (2016) 41:135–43. doi: 10.4103/0971-6203.181638 PMC4871003 27217626

[2] Chao P-J Tsai I-H Huang C-C Lin C-H Shieh C-S Hsieh Y-W. Radiation-induced secondary cancer risk assessment in patients with lung cancer after stereotactic body radiotherapy using the CyberKnife M6 system with lung-optimized treatment. Front Bioengineering Biotechnol. (2020) 8:306. doi: 10.3389/fbioe.2020.00306 PMC7223476 32457880

[3] Ianiro A Infusino E D’Andrea M Marucci L Farneti A Sperati F. Multiple brain metastases radiosurgery with cyberKnife device: dosimetric comparison between fixed/iris and multileaf collimator plans. J Med Phys. (2023) 48:120–8. doi: 10.4103/jmp.jmp_82_22 PMC10419749 37576098

[4] Lan J-H Shieh C-S Liu C-H Cho I-C Tsai I-H Chen L-C. Plan quality and secondary cancer risk assessment in patients with benign intracranial lesions after radiosurgery using the CyberKnife M6 robotic radiosurgery system. Sci Rep. (2019) 9:9953. doi: 10.1038/s41598-019-46133-8 31289294 PMC6616465

[5] Vellayappan BA McGranahan T Graber J Taylor L Venur V Ellenbogen R. Radiation necrosis from stereotactic radiosurgery—how do we mitigate? Curr Treat options Oncol. (2021) 22:57. doi: 10.3389/fonc.2018.00395 34097171

[6] Ma C-MC. Physics and dosimetric principles of SRS and SBRT. Mathews J Cancer Sci. (2019) 4:1–16. doi: 10.30654/MJCS.10022

[7] Nabavi M Nedaie HA Salehi N Naderi M. Stereotactic radiosurgery/radiotherapy: A historical review. Iranian J Med Phys. (2014) 11:156–67. doi: 10.22038/IJMP.2014.2621

[8] Mahdavi SR Tutuni M Farhood B Nafisi N Ghasemi S Mirzaee H. Measurement of peripheral dose to the pelvic region and the associated risk for cancer development after breast intraoperative electron radiation therapy. J Radiological Prot. (2019) 39:278. doi: 10.1088/1361-6498/aafdc8 30634170

[9] Kerckhove N Collin A Condé S Chaleteix C Pezet D Balayssac D. Long-term effects, pathophysiological mechanisms, and risk factors of chemotherapy-induced peripheral neuropathies: a comprehensive literature review. Front Pharmacol. (2017) 8:86. doi: 10.3389/fphar.2017.00086 28286483 PMC5323411

[10] Bezak E Takam R Yeoh E Marcu LG. The risk of second primary cancers due to peripheral photon and neutron doses received during prostate cancer external beam radiation therapy. Physica Med. (2017) 42:253–8. doi: 10.1016/j.ejmp.2017.02.018 28302493

[11] Delaby N Bellec J Bouvier J Jouyaux F Perdrieux M Castelli J. CyberKnife^®^ M6™: Peripheral dose evaluation for brain treatments. Physica Med. (2017) 37:88–96. doi: 10.1016/j.ejmp.2017.04.015 28535920

[12] Jang SY Lalonde R Ozhasoglu C Burton S Heron D Huq MS. Dosimetric comparison between cone/Iris-based and InCise MLC-based CyberKnife plans for single and multiple brain metastases. J Appl Clin Med Phys. (2016) 17:184–99. doi: 10.1120/jacmp.v17i5.6260 PMC5874093 27685124

[13] Schneider U Walsh L. Risk of secondary cancers: Bridging epidemiology and modeling. Physica Med. (2017) 42:228–31. doi: 10.1016/j.ejmp.2017.03.011 28363341

[14] Schneider U. Modeling the risk of secondary Malignancies after radiotherapy. Genes. (2011) 2:1033–49. doi: 10.3390/genes2041033 PMC3927608 24710304

[15] Tzikoulis V Gkantaifi A Alongi F Tsoukalas N Saraireh HH Charalampakis N. Benign intracranial lesions-radiotherapy: an overview of treatment options, indications and therapeutic results. Rev Recent Clin trials. (2020) 15:93–121. doi: 10.2174/1574887114666191111100635 31713498

[16] Hernandez V Hansen CR Widesott L Bäck A Canters R Fusella M. What is plan quality in radiotherapy? The importance of evaluating dose metrics, complexity, and robustness of treatment plans. Radiotherapy Oncol. (2020) 153:26–33. doi: 10.1016/j.radonc.2020.09.038 32987045

[17] Patel G Mandal A Choudhary S Mishra R Shende R. Plan evaluation indices: a journey of evolution. Rep Pract Oncol Radiotherapy. (2020) 25:336–44. doi: 10.1016/j.rpor.2020.03.002 PMC7082629 32210739

[18] Hamzah R Deevband MR Ghorbani M Khosravi M Pour FS Tavakoli M. Incidence risk assessment of secondary cancer due to radiotherapy of women with rectal cancer using BEIR VII, EPA, and ICRP models. Rep Pract Oncol Radiotherapy. (2023) 28:517–81. doi: 10.5603/rpor.96870 PMC10764039 38179292

[19] Kim J Seol Y Jang HS Kang Y-N. Spinal stereotactic body radiotherapy (SBRT) planning techniques. Ionizing Non-ionizing Radiat. (2020) 130–137. doi: 10.5772/intechopen.83515

